# Supplementary material for: Defining the optimal animal model for translational research using gene set enrichment analysis
Source: EMBO Mol Med. 2016 Jun 16;8(8):831–8. doi: 10.15252/emmm.201506025 (PMC4967938; doi:10.15252/emmm.201506025)
Supplement: Supplementary file 1 — Appendix [file EMMM-8-831-s001.pdf]

# APPENDIX

## Defining the optimal animal model for translational research using gene set enrichment analysis

Christopher Weidner, Matthias Steinfath, Elisa Opitz, Michael Oelgeschläger, Gilbert Schönfelder

**Table of contents** **Page**


- Appendix Figure S1 2-3
- Appendix Figure S2 4-5
- Appendix Table S1 6
- Appendix References 7

GSE37069

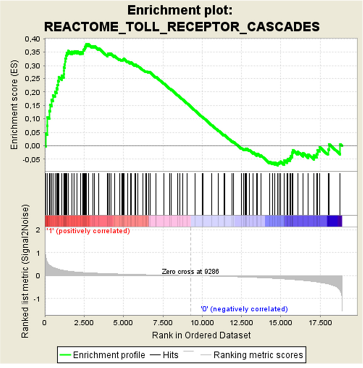


Normalized Enrichment Score = 1.50
Nominal *p* value = 0.004
FDR *q* value = 0.12

GSE36809

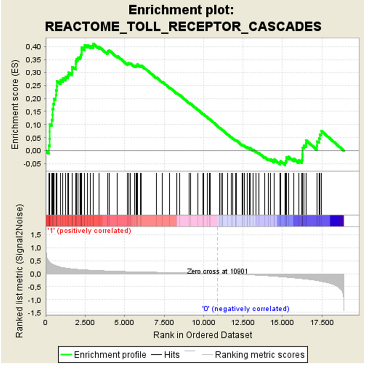


Normalized Enrichment Score = 1.70
Nominal *p* value = 0.0
FDR *q* value = 0.05

GSE3284

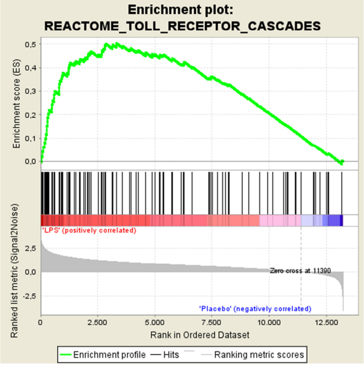


Normalized Enrichment Score = 1.67
Nominal *p* value = 0.0
FDR *q* value = 0.007

GSE9960

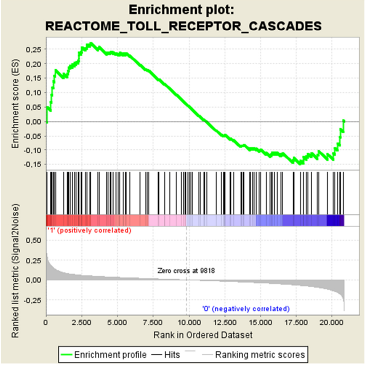


Normalized Enrichment Score = 1.11
Nominal *p* value = 0.25
FDR *q* value = 0.53

GSE13015

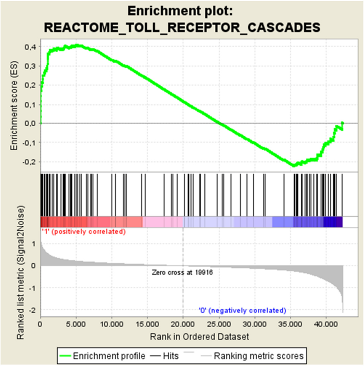


Normalized Enrichment Score = 1.67
Nominal *p* value = 0.0
FDR *q* value = 0.03

GSE13904

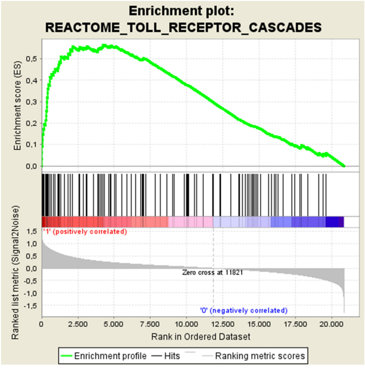


Normalized Enrichment Score = 2.14
Nominal *p* value = 0.0
FDR *q* value = 0.0

**Appendix Figure S1 (continued on next page).**

GSE28750

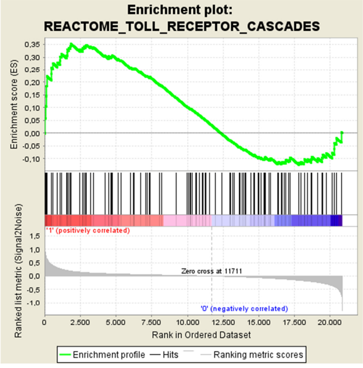


Normalized Enrichment Score = 1.33
Nominal *p* value = 0.05
FDR *q* value = 0.23

GSE20524

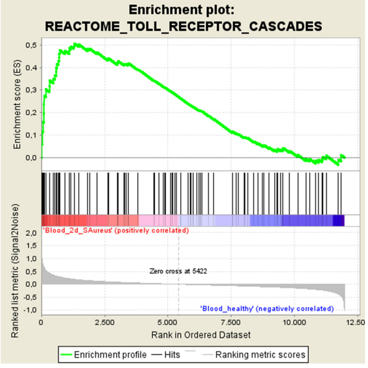


Normalized Enrichment Score = 1.90
Nominal *p* value = 0.0
FDR *q* value = 0.003

GSE19668

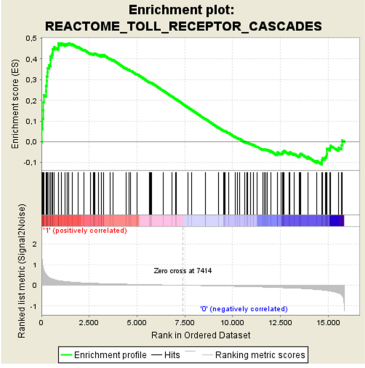


Normalized Enrichment Score = 1.71
Nominal *p* value = 0.0
FDR *q* value = 0.02

GSE5663 (CLP)

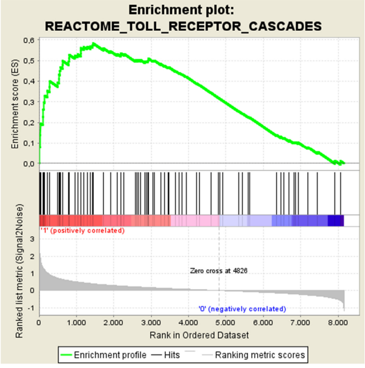


Normalized Enrichment Score = 1.75
Nominal *p* value = 0.0
FDR *q* value = 0.02

GSE5663 (CLPmild)

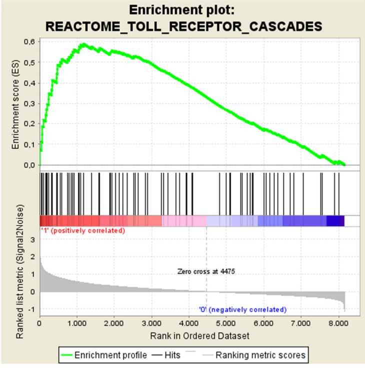


Normalized Enrichment Score = 1.80
Nominal *p* value = 0.0
FDR *q* value = 0.008

**Appendix Figure S1. Enrichment plots of the Toll receptor cascade (Reactome) pathway for datasets with high correlation.**

Enrichments plot and statistics were generated with the GSEA tool.

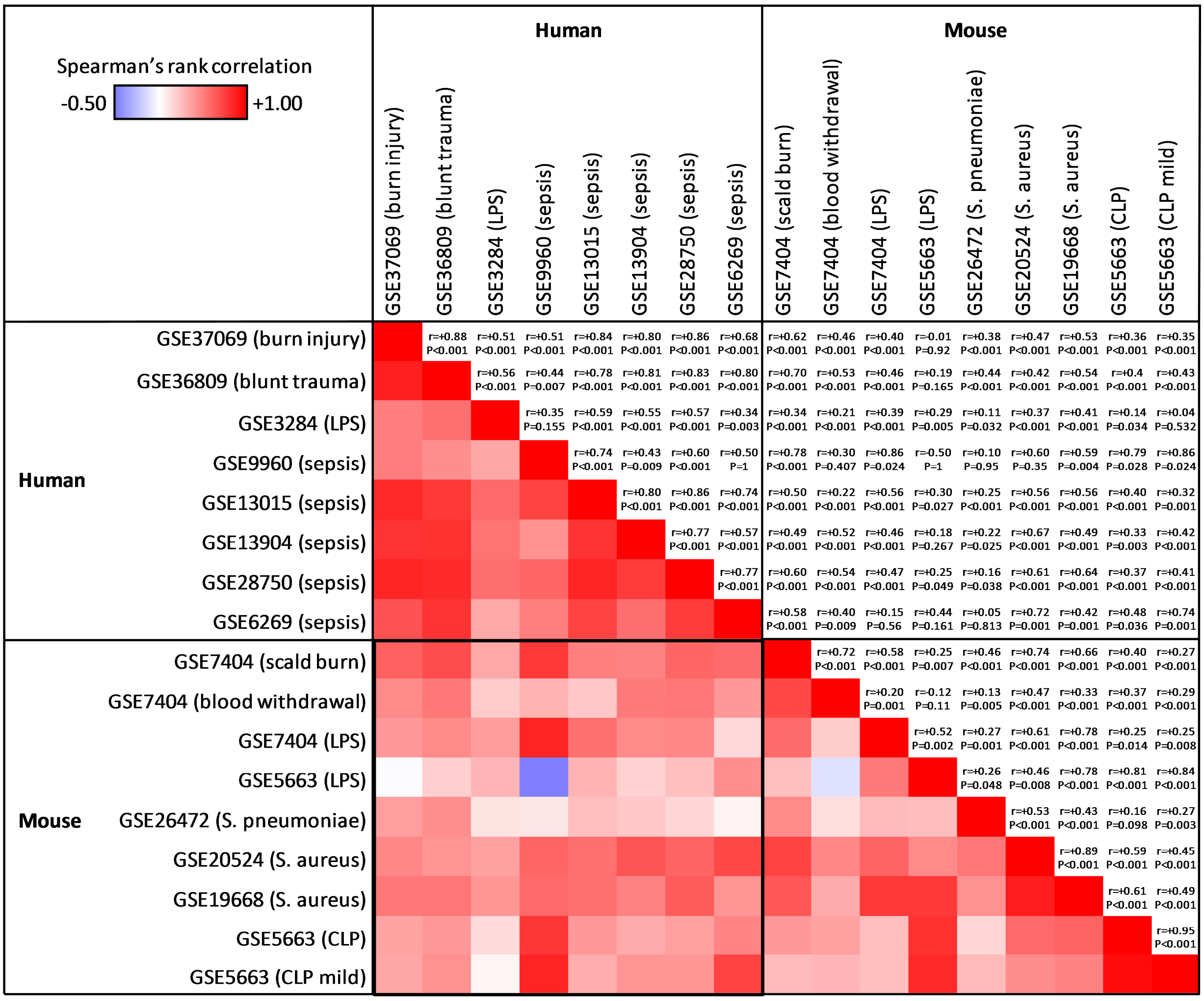


**Appendix Figure S2. Correlations of single-gene changes among human and mouse inflammatory studies.**

Spearman's rank correlation (r) was calculated based on log fold change values for differentially expressed genes according to the data selection strategy proposed by Takao et al. (Takao & Miyakawa, 2015). For correlation analyses between data sets the genes were selected as follows: fold change ratio > 2.0 or < 0.5 in human diseases, fold change ratio > 1.2 or < 0.83 in mouse models, (uncorrected) *P* value < 0.05 in *both* conditions of that comparison (note that correction of *P* values for multiple hypothesis testing could not be used since this would have led to some data sets with no differentially expressed genes at all). Most of the studies showed statistically significant correlations. Note that the gene selection criteria only include overlapping differentially

expressed genes and exclude genes that were differentially expressed in only one species. This single-gene approach thus tends to overestimate the correlation between human and mouse and allows no clear estimation of the best correlating mouse models.

**Appendix Table S1**

Transcriptomic studies used for model assessment on severe acute inflammatory diseases.

| Species | GEO accession | Stimulus type | Time after sampling |
|---|---|---|---|
| Human | GSE37069 | burn injury | 4 days |
| Human | GSE36809 | blunt trauma | 14 days |
| Human | GSE3284-GPL96 | LPS (2 ng/kg) i.v. | 6 hours |
| Human | GSE9960 | Septic patients | unspecified |
| Human | GSE13015 | Septic patients | unspecified |
| Human | GSE13904 | Septic patients | 1 day |
| Human | GSE28750 | Septic patients | unspecified |
| Human | GSE6269-GPL96 | Septic patients | unspecified |
| Mouse | GSE7404 | Scald burn | 7 days |
| Mouse | GSE7404 | Blood withdrawal | 1 day |
| Mouse | GSE7404 | LPS (10 ng) i.p. | 2 hours |
| Mouse | GSE5663 | LPS (20 mg/kg) i.p. | 24 hours |
| Mouse | GSE26472 | S. pneumoniae intratracheal | 6 hours |
| Mouse | GSE20524 | S. aureus i.v. | 2 days |
| Mouse | GSE19668 | S. aureus i.p. | 4 hours |
| Mouse | GSE5663 | CLP | 24 hours |
| Mouse | GSE5663 | CLP (mild) | 24 hours |

## Appendix References

Takao K, Miyakawa T (2015) Genomic responses in mouse models greatly mimic human inflammatory diseases. Proc Natl Acad Sci U S A 112: 1167-72
