# Supplementary material for: Defining the optimal animal model for translational research using gene set enrichment analysis
Source: EMBO Mol Med. 2016 Jun 16;8(8):831–8. doi: 10.15252/emmm.201506025 (PMC4967938; doi:10.15252/emmm.201506025)
Supplement: Supplementary file 2 — Expanded View Figures PDF [file EMMM-8-831-s002.pdf]

Expanded View Figures

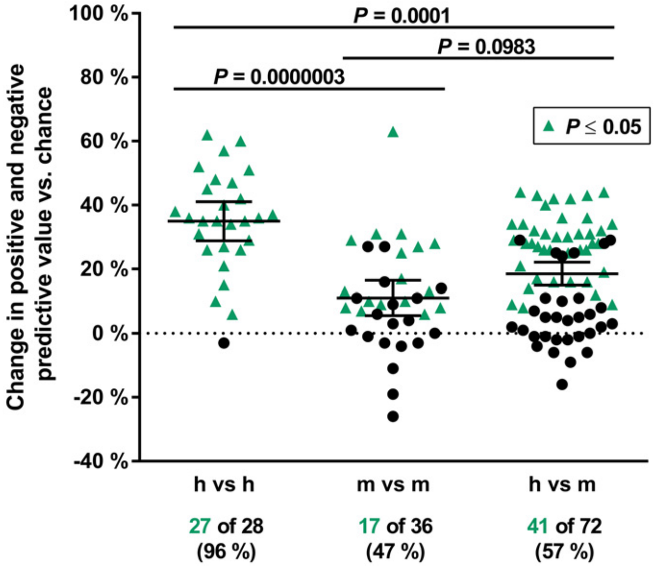

Figure EV1. Identification of inflammatory mouse models that show high correlations with human diseases.
The regulation of inflammatory pathways was assessed by gene set enrichment analysis (GSEA) using unfiltered gene expression data from 8 human and 9 mouse studies. Significantly regulated pathways were compared between two datasets from human (h) and/or mouse (m) studies. The degree of pathway overlap was calculated as the mean predictive values between these two datasets (see Fig 1A). The difference compared to expectation by chance (dotted line) is depicted as a change in the positive and negative predictive values relative to random overlap. Studies that revealed pathway overlap significantly greater than that expected by chance ($P \leq 0.05$) are labeled with green triangles. The numbers of significantly correlated studies are given below the datasets. Lines indicate the mean ± 95% confidence interval. The *P* values for each pair of datasets were calculated using a chi-squared test, and the *P* values for the comparison of species effects were calculated using the Kruskal–Wallis test followed by Dunn's multiple comparisons test and Bonferroni correction.

© 2016 The Authors
