# Supplementary material for: Defining the optimal animal model for translational research using gene set enrichment analysis
Source: EMBO Mol Med. 2016 Jun 16;8(8):831–8. doi: 10.15252/emmm.201506025 (PMC4967938; doi:10.15252/emmm.201506025)
Supplement: Supplementary file 3 — Dataset EV1 [file EMMM-8-831-s003.zip › Dataset_EV1/Readme.rtf]

This file contains the gene sets used for the GSEA analyses. These inflammatory pathways (95 in total) were manually downloaded from the BioCarta, Reactome and KEGG databases. For GSEA this file can be used as GMT (Gene Matrix Transposed) format.
